# Supplementary material for: Unreliability of Clinical Prediction Rules to Exclude without Echocardiography Infective Endocarditis in Staphylococcus aureus Bacteremia
Source: J Clin Med. 2022 Mar 9;11(6):1502. doi: 10.3390/jcm11061502 (PMC8955153; doi:10.3390/jcm11061502)
Supplement: Supplementary file 1 [file jcm-11-01502-s001.zip › jcm-1578367-supplementary.pdf]

Table S1: Items evaluated by PREDICT (5-day model), POSITIVE and VIRSTA scores.

ITEM		PREDICT (5-DAY MODEL)	POSITIVE	VIRSTA
**ACQUISITION**	Community	2 points	-	2 points
	Healthcare-associated	1 point	-	
	Nosocomial	0 point	-	0 points
**CARDIAC DEVICES**	Prosthetic valve	-	5 points for any of them	4 points any of them
	Pacemaker	3 points		
	Defibrillator	2 points		
**PERSISTENT BACTEREMIA**		2 points (>72 hours)	-	3 points (>48 hours)
**TIME TO POSITIVITY**	Less than 9 hours	-	5 points	-
	9-11 hours		3 points	
	11-13 hours		2 points	
	More than 13 hours		0 points	
**PREEXISTING NATURAL CARDIAC PREDISPOSITION**		-	5 points*	3 points
**INTRAVENOUS DRUG ABUSE**		-	3 points	4 points
**VASCULAR PHENOMENA/EMBOLI**		-	6 points	5 points
**PREVIOUS INFECTIVE ENDOCARDITIS**		-	-	4 points**
**MENINGITIS**		-	-	5 points
**VERTEBRAL OSTEOMYELITIS**		-	-	2 points
**SEVERE SEPSIS OR SHOCK**		-	-	1 points
**C-REACTIVE PROTEIN >190MG/L**		-	-	1 points
**POSSIBLE PUNCTUATION**		0-7	0-19	0-20
**CUT-OFF POINTS (HIGH RISK)**		Greater than 1 point	Greater than 4 points	Greater than 2 points

**In POSITIVE score, cardiac device, preexisting natural valve disease and other cardiac predisposition are considered as the same item: "predisposing heart disease"*

***In VIRSTA score, previous infective endocarditis and cardiac devices are considered as the same item: "permanent intracardiac device or previous infective endocarditis"*

Table S2: Evaluation of alternative cut-off points of different clinical prediction rules to identify IE among SAB patients. IE: infective endocarditis. SAB: Staphylococcus aureus bacteriemia. Sens: sensitivity. Spec: Specificity. PPV: Positive Predictive Value. NPV: Negative Predictive Value. PLR: Positive likelihood-ratio. NLR: Negative likelihood-ratio. AUC: Area under de curve.

Score	Cut-off point	Sens.	Spec.	PPV	NPV	NLR	PLR
**PREDICT (5-day model)**	> 0 points	90.0%	31.0%	15.9%	95.6%	0.32	1.30
**POSITIVE**	> 0 points	92.0%	35.6%	16.8%	96.9%	0.22	1.43
	> 3 points	84.0%	50.6%	19.4%	95.7%	0.32	1.70
**VIRSTA**	> 0 points	94.0%	24.0%	14.9%	96.5%	0.25	1.24
	> 1 point	94.0%	33.6%	16.7%	97.5%	0.18	1.42

Table S3: Individual characteristics of patients identified as low risk of IE by means of VIRSTA score but who finally were diagnosed of IE. IE: infective endocarditis. CIED: Cardiac implantable electronic device, PVC: peripheral venous catheter. ATB: antibiotic. TTE: transthoracic echocardiography, TEE: transesophageal echocardiography

VARIABLE	Patient 1	Patient 2	Patient 3	Patient 4
Age (YEARS)	91	91	72	75
Sex	Male	Male	Female	Female
Charlson index	1	0	7	0
Chronic heart failure	No	No	Yes	No
Natural cardiac valve disease	No	No	No	No
Prosthetic heart valve disease	No	No	No	No
CIED	No	No	No	No
Hemodialysis	No	No	No	No
Liver cirrhosis	No	No	No	No
Intravenous drug user	No	No	No	No
Previous IE	No	No	No	No
Acquisition	Nosocomial	Nosocomial	Nosocomial	Community
Primary source	PVC related	PVC related	Unknown*	Unknown
Symptoms duration prior to ATB	48 hours	24 hours	24 hours	48 hours
Sepsis/shock	No	No	No	No
Fever defervescence within 48h	Yes	Yes	Yes	Yes
Septic emboli	No	No	No	No
Thrombophlebitis	No	No	No	No
Acute cardiac failure	No	No	No	No
Time to positivity	18 hours	17 hours	13 hours	16 hours
Persistent bacteriemia	No	No	No	No
Early source control (if necessary)	Yes	Yes	Yes*	-
TTE	Positive	Positive	Negative	Not done
TEE	Not done	Not done	Positive	Positive
Outcome	IE-related death	IE-related death	Alive No relapse	Alive No relapse
PREDICT score classification	Low risk	Low risk	Low risk	High risk
POSITIVE score classification	Low risk	Low risk	Low risk	Low risk

**Patient 3 had a central venous catheter which was presumed to be the probable source of infection, although it could not be demonstrate. The catheter was retired 1 week previously to symptoms onset.*

Table S4: Univariate analysis of 30-day mortality in patients with no diagnosed with infective endocarditis and who survived the first 48 hours after the index blood culture extraction. Qualitative variables are expressed as percentage (absolute number) and compared by means of chi-square (or Fisher exact test when necessary). Quantitative variables are expressed as median (interquartile range) and compared by means of Mann-Whitney's U. CIED: Cardiac implantable electronic device. SAB: *Staphylococcus aureus* bacteriemia.

Variable		Total (n=335)	Survivor (n=281)	Non survivor (n=54)	p	Missing
**Demographic and comorbidity**						
**Age**		70 (56-81)	68 (54-80)	77 (64-90)	<0.001	0
**Sex (female)**		31.9% (107)	32.0% (90)	31.5% (17)	1.000	0
**Charlson index**		2 (1-5)	2 (0-4)	4 (2-6)	0.001	2
**Age-adjusted Charlson index**		5 (3-7)	5 (2-7)	7 (5-8)	<0.001	2
**Arterial hypertension**		52.8% (177)	50.9% (143)	63.0% (34)	0.136	0
**Diabetes mellitus**		27.8% (93)	28.5% (80)	24.1% (13)	0.619	0
**Chronic heart failure**		27.5% (92)	25.3% (71)	38.9% (21)	0.046	0
**Ischemic heart disease**		17.3% (58)	15.7% (44)	25.9% (14)	0.078	0
**Natural cardiac valve disease**		14.3% (48)	12.8% (36)	22.2% (12)	0.088	0
**Prosthetic heart valve disease**		3.0% (10)	2.8% (8)	3.7% (2)	0.667	0
**CIED**		4.8% (16)	5.3% (15)	1.9% (1)	0.334	0
**Chronic renal failure**		21.5% (72)	19.9% (56)	29.6% (16)	0.146	0
**Hemodialysis**		6.9% (23)	7.2% (20)	5.6% (3)	0.789	0
**Liver cirrhosis**		3.0% (10)	2.5% (7)	5.6% (3)	0.209	0
**Solid organ malignancy**		22.2% (74)	21.8% (61)	24.1% (13)	0.722	1
**Parenteral drug user**		0.9% (3)	1.1% (3)	0	1.000	1
**Clinical presentation**						
**Acquisition**	**Nosocomial**	53.1% (178)	53.7% (151)	50.0% (27)	Ref.	0
	**Healthcare associated**	17.6% (59)	16.4% (46)	24.1% (13)	0.224	
	**Community**	29.3% (98)	29.9% (84)	25.9% (14)	0.843	
**Source of infection**	**Catheter-related**	35.5% (119)	37.0% (104)	27.8%% (15)	ref	0
	**Primary-Unknown**	24.5% (80)	20.8% (57)	43.8% (23)	0.001	
	**Other**	40.6% (136)	42.7% (120)	29.6% (16)	0.837	
**Fever**		89.8% (300)	88.9% (249)	94.4% (51)	0.325	1
**Sepsis/septic shock**		23.1% (77)	18.9% (53)	44.4% (24)	<0.001	1
**Fever defervescence within 72 hours**		82.3% (276)	83.2% (234)	72.2% (39)	0.305	29
**Septic emboli**		9.0% (30)	9.3% (26)	7.4% (4)	0.799	0
**Acute kidney injury**		36.0% (119)	32.5% (90)	53.7% (29)	0.003	4
**Acute cardiac failure**		15.0% (50)	12.5% (35)	27.8% (15)	0.007	2
**Pitt's bacteremia score**		0 (0-1)	0 (0-1)	1 (0-3)	<0.001	1
**SOFA**		2 (0-4)	1 (0-3)	4 (2-5)	<0.001	2
**Microbiology**						
**Time to positivity**		12 (10-16)	12 (10-16)	12 (9-17)	0.423	0
**Persistent bacteriemia**		26.9% (71)	25.0% (58)	40.6% (13)	0.087	71
**Meticilin-resistant SAB**		20.6% (69)	18.9% (53)	29.6% (16)	0.057	0
**Diagnostic work-up**						
**TTE and/or TEE**		72.0% (237)	75.9% (211)	51.0% (26)	<0.001	6
**TTE**		62.3% (207)	66.4% (186)	40.4% (21)	0.001	3
**TEE**		28.0% (11)	29.1% (81)	21.6% (11)	0.311	6
**PET-CT**		8.1% (27)	8.9% (25)	3.8% (2)	0.279	3
**Complicated SAB**		28.1% (93)	26.1% (73)	39.2% (20)	0.042	4
**VIRSTA score (high risk)**		49.0% (164)	45.2% (127)	68.5% (37)	0.002	0
**Source control necessary and not performed**		11.3% (38)	10.7% (30)	14.8% (8)	0.252	0
